# Molecular Rewiring of the Jasmonate Signaling Pathway to Control Auxin-Responsive Gene Expression

**DOI:** 10.3390/cells9030641

**Published:** 2020-03-06

**Authors:** Ning Li, Linggai Cao, Wenzhuo Miu, Ruibin Cao, Mingbo Peng, Wenkai Wan, Li-Jun Huang

**Affiliations:** 1 State Key Laboratory of Cultivation and Protection for Non-Wood Forest Trees, Ministry of Education, College of Forestry, Central South University of Forestry and Technology, Changsha 410004, China; nli@csuft.edu.cn (N.L.); alic5e@163.com (W.M.);; 2 Key Laboratory of Saline-Alkali Vegetation Ecology Restoration (SAVER), Ministry of Education, College of Life Science, Northeast Forestry University, Harbin 150040, China; caolinggai@126.com

**Keywords:** jasmonic acid, auxin, protoplast, signaling pathway, gene expression

## Abstract

The plant hormone jasmonic acid (JA) has an important role in many aspects of plant defense response and developmental process. JA triggers interaction between the F-box protein COI1 and the transcriptional repressors of the JAZ family that leads the later to proteasomal degradation. The Jas-motif of JAZs is critical for mediating the COI1 and JAZs interaction in the presence of JA. Here, by using the protoplast transient gene expression system we reported that the Jas-motif of JAZ1 was necessary and sufficient to target a foreign reporter protein for COI1-facilitated degradation. We fused the Jas-motif to the SHY2 transcriptional repressor of auxin signaling pathway to create a chimeric protein JaSHY. Interestingly, JaSHY retained the transcriptional repressor function while become degradable by the JA coreceptor COI1 in a JA-dependent fashion. Moreover, the JA-induced and COI1-facilitated degradation of JaSHY led to activation of a synthetic auxin-responsive promoter activity. These results showed that the modular components of JA signal transduction pathway can be artificially redirected to regulate auxin signaling pathway and control auxin-responsive gene expression. Our work provides a general strategy for using synthetic biology approaches to explore and design cell signaling networks to generate new cellular functions in plant systems.

## 1. Introduction

Being autotrophic and fixed in space, plants are under relentless challenges by numerous environmental stresses. Plant development and growth are superplastic in response to changing environments. Upon detection of dangers, plants carry out multiple layers of response to coordinate development and defense, orchestrated by an elegantly organized signaling network of phytohormones, such as auxin that acts as a general coordinator of plant development and jasmonic acid that mainly regulates plant response to environmental stimuli [1,2,3].

Over recent years, mounting lines of studies revealed extensive crosstalk, either synergistic or antagonistic, among different hormonal signaling pathways [4]. For instance, JA can directly activate the expression of genes that are involved in auxin biosynthesis to regulate tissue regeneration and lateral root formation in *Arabidopsis* [5,6,7]. While, on the other hand, auxin stimulates adventitious root formation on *Arabidopsis* hypocotyls by inducing expression of genes encoding enzymes that are involved in inactivating JA or reducing JA accumulation [8]. However, the molecular mechanisms underlying signaling crosstalk are remain poorly understood. Elucidating hormone signaling pathways is not only important for understanding fundamental questions in plant biology but also crucial for breeding programs to enhance stress tolerance and improve yield performance in agricultural crops. For instance, exogenously applied methyl jasmonate effectively improves the drought tolerance in soybean (*Glycine max* L. Merrill) [9], wheat (*Triticum aestivum* L.) [10], and rice (*Oryza sativa* subsp. *japonica*) [11]. Recently, Zhang et al. succeeded in engineering the JA coreceptor COI1 that allows for endogenous JA signaling but with reduced sensitivity to pathogen produced phytotoxin [12]. Transgenic *Arabidopsis* plants expressing this modified COI1 show better resistance to disease-causing pathogens such as *Pseudomonas syringae*.

JA is a relatively newly discovered plant hormone class comprised of lipid-derived small-molecules [13,14]. JA regulates plant defense responses against biotic and abiotic stresses, and affects plant development as well [15,16,17]. Our present knowledge about JA signaling pathway are largely from studies of the model plant *Arabidopsis thaliana* [13,18,19,20]. JA is biosynthesized from membrane lipid α-linolenic acid via the octadecanoid pathway [21]. Once synthesized, JA is further adenylated at the carboxylic end by a JA-amido conjugate synthase to form the final bioactive derivative, jasmonoyl-isoleucine (JA-Ile) [22,23]. JA-Ile is perceived by a coreceptor complex consisting of the F-box protein CORONATINE INESENSTIVE 1 (COI1) and the JASMONATE ZIM-DOMAIN (JAZ) family of transcriptional repressors [24,25,26]. COI1 is the substrate-recognition component of the Skp1/Cullin/F-box protein (SCF) ubiquitin E3 ligase complex, SCF^COI1^ [27,28].

Under normal growth conditions when JA levels are low, JA-responsive genes are actively repressed by the JAZ repressors which physically associate with JA-network MYC transcription factors. *Arabidopsis* genome encodes 13 JAZ repressors [29]. JAZs belong to the plant-specific TIFY protein family which is defined by the presence of TIFY-motif within a larger conserved ZIM domain [30,31]. The TIFY-motif is involved in mediating the JAZ interaction with Novel Interactor of JAZ (NINJA). NINJA serves as an adaptor protein to recruit the TOPLESS (TPL) and TPL-related (TPR) transcriptional corepressors [32]. TPL and TPRs are members of the evolutionarily conserved Groucho/Tup1-type corepressor family that coordinate the formation of transcriptional repression complexes with histone-modifying enzymes such as histone deacetylases (HDACs), resulting in a suppressive chromatin state inaccessible to transcription machineries [33,34]. Therefore, the JAZ-NINJA-TPL complex represses MYC transcriptional activity through recruitment of HDACs in order to restrain JA response. Upon pathogen or insect attack when JA concentration reaches a threshold in the cell, JA directly triggers COI1 binding to JAZ proteins, resulting in the ubiquitination and subsequent degradation of JAZ repressors via the 26S proteasome thereby releasing MYC-dependent transcription of JA-regulated genes. Protein ubiquitination is an important post-translational process that is regulated by at least three main families of enzymes: ubiquitin activating enzymes (E1), ubiquitin conjugating enzymes (E2) and ubiquitin ligases (E3) [35]. Protein substrates are specifically recruited by the E3 ligases to the proximity of E2, which attaches the ubiquitin moiety to a lysine residue in the targets [36]. Targets may be monoubiquitinated by single ubiquitin in one position or several positions, or polyubiquitinated by a chain of ubiquitin polymers. So far, ubiquitination sites in JAZ proteins have not been identified. In addition to containing a central ZIM domain, JAZ proteins also contain a highly conserved C-terminal JA associated- (Jas-) motif [29]. The Jas-motif was found to be the minimal region being indispensable and adequate to mediate the JAZ-COI1 interaction.

Interestingly, the growth hormone auxin and the defense hormone JA share mechanistically conserved ligand perception and signal transduction mechanisms [37]. Auxin is perceived by a protein complex containing the F-box protein Transport Inhibitor Response 1 (TIR1) and the auxin/indole-3-acetic acid (Aux/IAA) transcriptional repressor proteins [38,39]. The Aux/IAA proteins directly interact with TPL corepressors through an ethylene response factor-associated amphiphilic repression (EAR) motif to form the Aux/IAA-TPL repressive complex that represses auxin response factors (ARFs) at the promoter region of auxin response genes [40]. In the presence of auxin, Aux/IAA repressors are ubiquitinylated by the SCF^TIR1^ complex [41]. IAA6 and IAA19 of the Aux/IAA family are conjugated with polymeric ubiquitin chains at multiple lysine residues [42]. Polyubiquitination of Aux/IAAs leads them to degradation by the 26S proteasome pathway. Degradation of Aux/IAA releases the transcriptional activity of ARF regulators.

Previously, we have reconstituted the core JA signaling pathway in transiently transformed protoplasts [43]. Analysis of the stability of a JAZ1-fLuc fusion protein Li et al. recapitulated the JA-induced degradation of JAZ1 repressor by COI1 and confirmed that the Jas-motif was important for this degradation. The protoplast transient gene expression system offers invaluable opportunities for studying subcellular protein localization, transcriptional regulation, genome editing, and plant synthetic biology [44,45]. Because of its flexibility and convenience, protoplast expression system has become a useful tool for analyzing cellular signaling mechanisms. Using a similar approach, we investigated the stability of a Jas-fLuc fusion protein in *Arabidopsis* protoplast cells and determined that the Jas-motif alone is necessary and sufficient to mark the nonrelated fLuc reporter to targeted protein degradation. This result further inspired attempts to artificially connect the JA signaling cascade to manipulate gene expression of the auxin pathway by tagging the Jas-motif to the Aux/IAA proteins. Our study provides the basis for generating further understanding of the molecular and cellular mechanisms of hormone action, which could eventually facilitate strategies for crop engineering to produce more and better foods.

## 2. Materials and Methods

### 2.1. Plant Materials and Cultural Conditions

*Arabidopsis thaliana* of the Columbia (Col-0) ecotype was used throughout this study. *Arabidopsis coi1* (Salk035548) [46] mutant seeds were surface sterilized by exposure to chloric gas produced from the mixture of 100 mL 12% sodium hypochlorite (Carl Roth, Karlsruhe, Germany) and 3.5 mL 37% Salzsäure (Carl Roth, Karlsruhe, Germany) in a sealed desiccator for at least 3 h. To select homozygous plants, the *coi1* seeds were sown on MS-agar plates (Duchefa, Haarlem, The Netherlands) containing 50 µM methyl jasmonate (Sigma-Aldrich, Darmstadt, Germany) [47]. *Arabidopsis* plants were germinated and grown in a growth chamber (Percival Scientific, Germany) under a 12-h-light/12-h-dark regime at 22/20 °C with 80–100 μmol photons m^−2^ s^−1^.

### 2.2. Molecular Cloning

Two types of reporter vector were used in this study: the protein-fusion reporter vector and the promoter-driven reporter vector. The GATEWAY (GW) destination vector *UBQ10pro*:HA-GW-fLuc [43] was used to clone protein-fusion reporter vectors. The Jas-motif coding sequence of JAZ1 (Jas) was obtained by polymerase chain reaction (PCR) using pDONR207-JAZ1 [43] as template with primer set JAS-gw-d1/JASnostop-gw-r1(All primer sequences are listed in Table 1). The Jas^AA^ mutant variant was amplified by PCR with primer set JASA-gw-d1/JASnostop-gw-r1. The Jas and Jas^AA^ PCR products flanked by the *attB* sequence were cloned into pDONR207 (Invitrogen, Carlsbad, CA, USA) according to the manufacturer’s instructions. The resulting entry clone pDONR207-Jas and -Jas^AA^ were recombined with the destination vector to produce the final protein-fusion reporter vectors *UBQ10pro*:Jas-fLuc and *UBQ10pro*:Jas^AA^-fLuc. The SHY2 gene coding sequence was obtained by PCR using pDONR207-SHY2 (pDONR207-IAA3) [48] as template with primer set SHY2-gw-d1/SHY2nostop-gw-r1. The PCR product flanked by the *attB* sequence were cloned into pDONR207, yielding the entry clone pDONR207-SHY2nostop. Three PCR reactions were used to obtain the JaSHY chimera coding sequence, one reaction used the pDONR207-Jas as template with primer set SeqL1/JASHY-r1 to amplify a Jas sequence that encloses a 5′-portion of SHY2 coding sequence. A second PCR reaction used pDONR207-SHY2nostop as template with primer set JASHY-d1/SeqL2 to amplify the SHY2 coding sequence that encloses a 3′-portion of Jas coding sequence. The PCR products from these two reactions contain complementary sequences that allow the products to anneal as template in a third PCR reaction with primer set SeqL1/SeqL2. The chimeric product containing the *attL* sequence was directly recombined with the destination vector to produce the protein-fusion reporter vector *UBQ10pro*:JaSHY-fLuc.

The GW destination vector pBGWFL7 [49] was used to clone promoter-driven reporter vector. The *DR5* promoter sequence was amplified from the *DR5*-GUS construct [50] with primer set DR5-gw-d1/DR5-gw-r1. The PCR product containing *DR5* promoter sequence was cloned into pDONR207, resulting entry clone pDONR207-DR5pro for recombing with the destination vector pBGWFL7 to produce the promoter-driven reporter vector *DR5pro*:fLuc.

The GW destination vector *UBQ10pro*:HA-GW [48] was used to clone effector vectors. The ARF7 gene coding sequences were obtained by PCR using *Arabidopsis* cDNA as template with primer set ARF7-gw-d1 and ARF7-gw-r1. The JaSHY chimera coding sequence was amplified using *UBQ10pro*:JaSHY-fLuc as template with primer set JAS-gw-d1/SHY2-gw-r1. The PCR products were cloned into pDONR207 resulting entry clones pDONR207-ARF7 and -JaSHY. pDONR207-ARF7, -SHY2 and -JaSHY were recombined with the destination vector to produce the effector vectors *UBQ10pro*:ARF7, *UBQ10pro*:SHY2, and *UBQ10pro*:JaSHY. Construction of the effector vectors *UBQ10pro*:COI1 and *UBQ10pro*:COI1^lrr13^ was described in [43].

### 2.3. Protoplast Transfection and Dual Luciferase Report Assay

Protoplast transfection and dual luciferase assay were performed as described previously in [43,51]. Briefly, 5 µg of protein-fusion reporter plasmids or promoter-driven reporter plasmids and 5 µg of effector plasmids were co-transfected. To normalize for the experimental variability, 1 µg of the reference plasmids *UBQ10pro*:rLuc [48] were added to each transfection. The empty effector plasmids *UBQ10pro*:HA [48] lacking the GATEWAY cassette were added, when necessary, to provide for equal amounts of total DNA in each transfection experiment. Luc activities were determined 16 h after transfection of protoplasts using the Dual-Luciferase Reporter Assay System (Promega, Mannheim, Germany) and the Centro XS^3^ LB 960 Microplate Luminometer from Berthold Technologies (Bad Wildbad, Germany).

## 3. Results and Discussion

### 3.1. The Jas-Motif Is Sufficient to Target Luciferase Reporter for Degradation

Previously, we developed a transient gene expression system in *Arabidopsis coi1* mutant protoplasts, by which the core JA signaling pathway was successful reconstituted [43]. The JAZ1 repressor of JA signaling pathway was degraded via the 26S proteasome in a JA-induced and COI1- facilitated manner in the protoplast reconstitution assay. The Jas-motif of JAZ1 protein (referred to as Jas) was required for the JA-dependent interaction with COI1 [29]. Substitution mutations at critical residues of Jas abolish COI1-JAZ1 interaction and confer JAZ1 protein resistant to JA [43].

**Figure 1:**
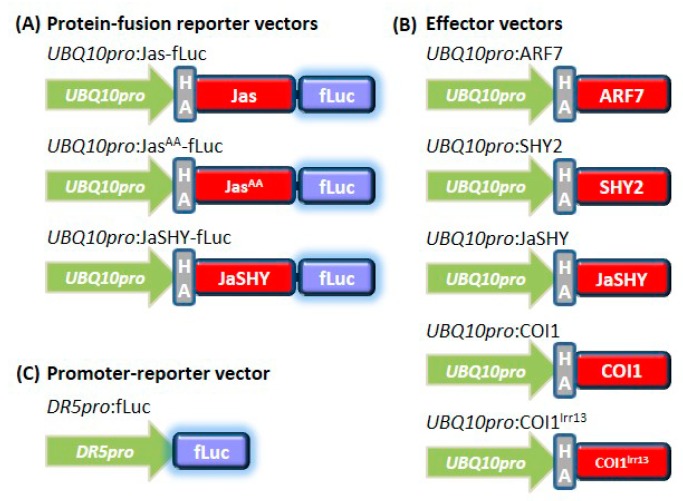
Scheme of vectors used for protoplast transient transfections in this study. (**A**) The protein-fusion reporter vectors. The coding sequences of wild-type and mutant Jas-motif and the JasSHY chimeric protein were fused in-frame to the N-terminus of firefly luciferase (fLuc) reporter gene. Expression of reporter proteins were under the control of the *UBQ10* promoter. (**B**) The effector vectors. Expression of SHY2, JaSHY chimera, COI1 and COI1 mutant effector proteins were placed under the control of the *UBQ10* promoter. All reporter and effector proteins contain a hemagglutinin (HA) epitope tag at the N-terminus. (**C**) The promoter-reporter vector. The auxin responsive *DR5* promoter sequence was fused to the firefly luciferase (fLuc) reporter gene. Not drawn to scale.

**Figure 2:**
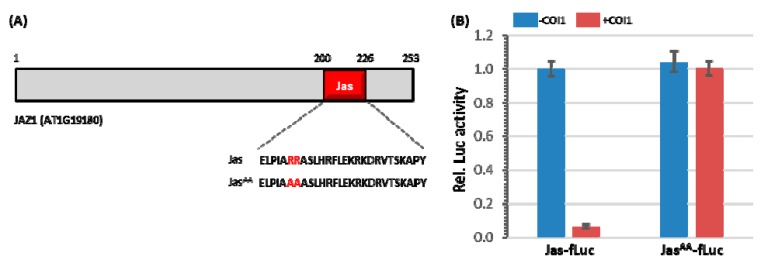
The Jas-motif of JAZ1 is sufficient to target fLuc for degradation. (**A**) Schematic and amino acid residues of JAZ1 Jas-motif. The red bar indicates residues of the Jas-motif with the sequence shown below. The red letters highlight wild-type residues and point mutations in Jas and Jas^AA^, respectively. (**B**) *Arabidopsis* leaf protoplasts prepared from *coi1* mutant plants were co-transfected with protein-fusion reporter plasmids encoding Jas-fLuc or Jas^AA^-fLuc under the control of *UBQ10* promoter and effector plasmid encoding COI1 under the control of *UBQ10* promoter. Firefly luciferase (fLuc) activities were normalized to *Renilla* luciferase (rLuc) activities. Reporter activities of Jas-fLuc without effector vector were set to one. Values represent means (±SE) of four independently transformed batches of protoplasts.

To determine whether Jas is not only necessary for JAZ1 degradation, but also sufficient for targeting a heterologous protein for degradation, the 27 aa Jas coding sequence was placed in frame upstream of firefly luciferase (fLuc) for translational fusion and the *Arabidopsis UBQ10* promoter was placed upstream of the fusion protein coding region, creating the protein-fusion reporter construct *UBQ10pro*:Jas-fLuc (Figure 1A). The protein-fusion reporter construct was transfected alone or together with the effector construct *UBQ10pro*:COI1 (Figure 1B). As shown in Figure 2B, the Jas-fLuc activity was dramatically decreased in the presence of COI1 in *coi1* mutant protoplasts. This result suggests that the Jas sequence that requires for JAZ1 degradation is capable of functioning as a transferable degradation signal targeting the fLuc reporter protein for proteolysis. In order to check whether the Jas-mediated fLuc degradation is JA dependent or not, the two critical arginine residues (R_205_R) of Jas required for COI1-JA-JAZ1 complex formation was exchanged into alanines (A_205_A) (Figure 2A), creating the Jas^AA^-fLuc reporter protein. Interestingly, these amino acid substitutions did not affect Jas^AA^-fLuc protein accumulation in *coi1* protoplasts but caused the reporter protein insensitive to COI1 (Figure 2B). This result indicates the importance of JA in conferring Jas-mediated fLuc degradation. Thus, we determined in *Arabidopsis* protoplasts that Jas alone is sufficient for JA-induced and COI1-mediated protein degradation, which could be used to develop a JA-inducible protein depletion system. Protein abundance reflects the balance between protein synthesis and protein degradation. The classical cycloheximide chase experiment in combination with MG132 treatment, however, is further required to confirm exclusively that the observed decrease of fLuc activity is indeed linked to the degradation of the fusion protein via the 26S proteasome pathway. Previously, Larrieu et al. fused the Jas-motif of JAZ9 (Jas9) to the VENUS variant of the yellow fluorescent protein (YFP) to generate a JA biosensor named Jas9-VENUS that allows quantitative detection of JA distribution in *Arabidopsis* with high spatiotemporal sensitivity [52]. The Jas-fLuc reporter has the potential to serve as a biosensor for nondestructive detection of the spatial and temporal JA distribution in vivo and in real time. 

In plants, the signaling pathway of JA strikingly resembles to that of auxin, in which the F-box protein, TIR1 of the auxin pathway or COI1 of the JA pathway, forms SCF^TIR1^ or SCF^COI1^ protein complex that recognizes the JAZ or Aux/IAA transcriptional repressors of respective pathways to regulate plant responses [37,53]. In the presence of auxin, TIR1 binds to Aux/IAA to facilitate ubiquitination and proteasomal degradation of Aux/IAA. The conserved Domain II (known as degron) of Aux/IAA proteins was determined to be the minimal sequence that is sufficient to mediate TIR1 and Aux/IAA interaction. The auxin-inducible protein degradation (AID) system was developed as a tool to conditionally control protein stability in nonplant systems [54,55,56,57]. Analogously, it is possible to develop a JA-inducible protein degradation (JID) system to control protein function in a tunable way.

### 3.2. The Jas-Tagging Leads the Aux/IAA Protein SHY2 to Be Degraded by the JA Coreceptor COI1

**Figure 3:**
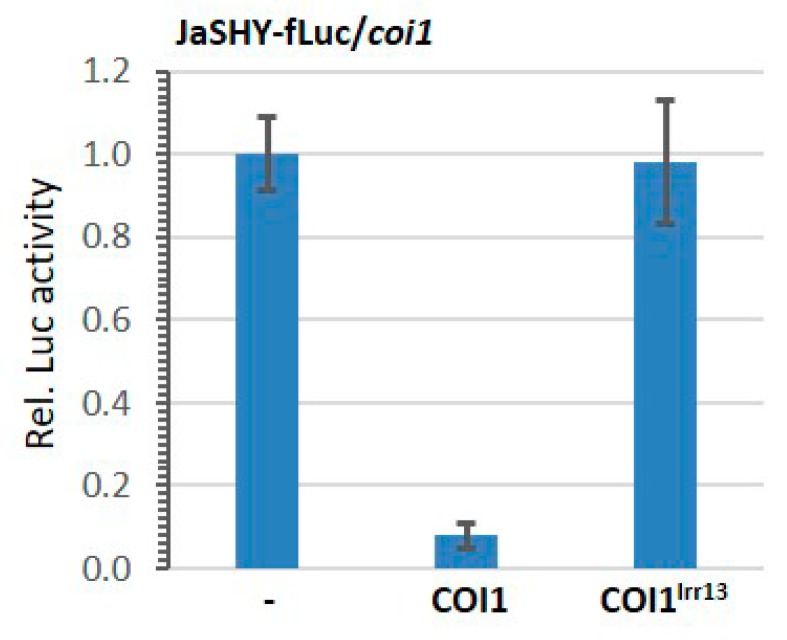
The expression of protein-fusion reporter JaSHY-fLuc was reduced by COI1 in *coi1* protoplasts. *Arabidopsis* leaf protoplasts prepared from *coi1* mutant plants were co-transfected with protein-fusion reporter plasmid encoding JaSHY-fLuc under the control of *UBQ10* promoter and effector plasmids encoding COI1 or COI1^lrr13^ under the control of *UBQ10* promoter. Firefly luciferase (fLuc) activities were normalized to *Renilla* luciferase (rLuc) activities. Reporter activities of JaSHY-fLuc without effector vector were set to one. Values represent means (±SE) of four independently transformed batches of protoplasts.

As a proof of concept to develop a functional JA-inducible protein degradation (JID) system, we fused Jas to the Aux/IAA protein SHORT HYPOCOTYL2 (SHY2, also known as IAA3) and placed the chimeric protein Jas-SHY2 (JaSHY for short) upstream of fLuc to create the JaSHY-fLuc fusion reporter. Expression of the fusion reporter was put under the control of the *UBQ10* promoter (*UBQ10pro*:JaSHY-fLuc, Figure 1A). SHY2, a canonical Aux/IAA protein from *Arabidopsis*, was selected for this assay because SHY2 is important in multiple auxin responses, as demonstrated by identification of an auxin response mutant *shy2* which showed a plethora of pleiotropic growth phenotypes, such as short wavy roots, enlarged cotyledons, short hypocotyls, and extremely dwarfed plants with curled leaves [58,59,60]. Again, JaSHY-fLuc was expressed alone or together with the effector COI1 in *coi1* protoplasts. Co-expression of COI1 strongly decreased the JaSHY-fLuc activity (Figure 3). Control experiments were performed using a mutant effector COI1^lrr13^. In order not to disrupt protein folding, critical residues of the COI1 13th LRR-motif (YMA_384_VYV) involved in JA-Ile binding were substituted with the motif SVL_378_YFC found in the structurally related auxin receptor TIR1 [43]. Since this mutant derivate cannot bind to JA, COI1^lrr13^ had no effect on JaSHY-fLuc reporter accumulation (Figure 3), demonstrating that Jas tag specifically recruited JaSHY-fLuc to SCF^COI1^. These results indicate that the COI1-mediated degradation of JaSHY-fLuc reporter protein is JA-dependent.

So far, by tagging Jas to SHY2, we were able to destroy a repressor of the auxin signaling pathway in a JA-inducible way. Despite this interest, fundamental questions remain regarding the potency and efficacy of JA-induced and Jas-mediated binding of SHY2 to COI1. And, particularly, whether a Jas tag at the N-terminus impinges SHY2 protein function requires further clarification. Our work only represented preliminary results of this concept in isolated protoplast cells.

### 3.3. Degradation of the JaSHY Chimeric Repressor Liberates ARF7 Transcription Activity

**Figure 4:**
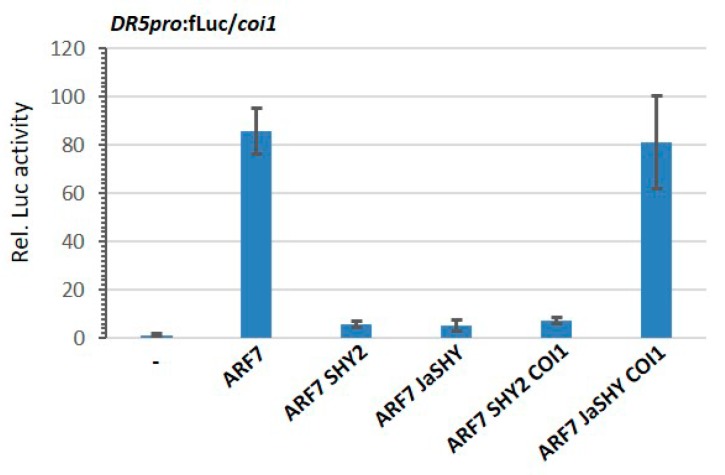
The ARF7 transcriptional activity was released upon JaSHY degradation by COI1. *Arabidopsis* leaf protoplasts prepared from *coi1* mutant plants were co-transfected with promoter-driven reporter plasmid *DR5pro*:fLuc and effector plasmids encoding ARF7, SHY2, JaSHY, or COI1 under the control of *UBQ10* promoter. Firefly luciferase (fLuc) activities were normalized to *Renilla* luciferase (rLuc) activities. Reporter activities of fLuc without effector vector were set to one. Values represent means (±SE) of four independently transformed batches of protoplasts.

Auxin signaling involves the activation of gene expression by a bevy of ARF factors that bind to canonical auxin response elements (AuxREs) in auxin-responsive gene promoters [50]. The Aux/IAA proteins negatively modulate auxin-regulated gene expression as transcriptional repressors through heterodimerization with the ARF transcription activators. The release of ARF repression in the presence of auxin by the proteasomal degradation of their cognate Aux/IAA repressors elicits a rapid transcriptional change [61]. The ARF7-SHY2 signaling module plays a crucial role in many aspects of plant growth and development [62,63,64]. Since by introducing a Jas-tag the SHY2 repressor become degradable by the JA coreceptor COI1 (Figure 3), we sought to rewire the JA signaling pathway to modulate expression of auxin-responsive gene. We first confirmed that in protoplasts ARF7 strongly activated the fLuc reporter gene under control of the synthetic auxin-responsive promoter *DR5* (*DR5pro*:fLuc; Figure 1C) as manifested by the increased fLuc bioluminescence (Figure 4). Both SHY2 and JaSHY chimeric repressor drastically repressed the ARF7-activated *DR5* promoter activity to the same level (Figure 4). However, by the addition of COI1, only JaSHY- but not SHY2-mediated repression was alleviated (Figure 4). These results indicate that the Jas-tag does not influence SHY2 repressive function and the specific removal of the JaSHY chimeric repressor by COI1 leads to the liberation of ARF7 transcriptional activity. Therefore, by tagging Jas to an auxin repressor, we could manipulate auxin-regulated gene expression in a JA-inducible and auxin-independent manner.

In general, the auxin signaling pathway plays an important role in plant development, whereas the JA signaling pathway primarily regulates plant defense response. Increasing lines of evidence showed that sophisticated crosstalk between phytohormone signaling pathways fine-tunes the action of those hormones [4]. The JA and auxin signal transduction pathways are mutually antagonistic [65].

Defense-activated JA signaling pathway inevitably compromises auxin-regulated developmental processes [66]. However, by introducing JA-inducible degron to the Aux/IAA repressors, it could link the JA signaling pathway to control gene expression downstream of Aux/IAA regulators and achieve both JA and auxin responses simultaneously.

## 4. Conclusions

**Figure 5:**
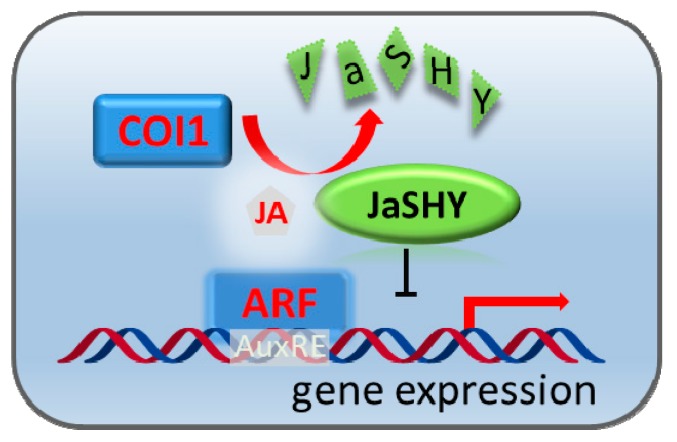
Schematic representation of jasmonate-induced, ARF7-mediated auxin responsive gene expression in protoplasts. In the absence of JA, the transcriptional activity of ARF7 is repressed by the chimeric protein JaSHY. In the presence of JA, JaSHY is degraded by COI1, leading to the liberation of ARF7 transcriptional activity.

This work showed that the Jas-motif of JAZ1 protein is not only required but also sufficient to target an unrelated protein such as fLuc to SCF^COI1^ for degradation in plant protoplasts. By fusing the Jas-motif to the SHY2 transcriptional repressor of auxin signaling pathway, we developed a rapid experimental system to rewire the JA signaling pathway to control auxin-responsive gene expression (Figure 5). To our knowledge, this is the first development of a JA-inducible protein degradation system and use of a plant hormone signaling pathway to control gene expression and potentially physiological response of another plant hormone. We envision that it even may be possible to employ this system to design crops that overcome the auxin- and JA-mediated growth-defense tradeoffs to grow substantially health and produce more food than the most productive varieties today.

## Figures and Tables

**Table 1:** Primer sequences used in this study.

Primer Symbol	Primer Sequence (5′-3′)
JAS-gw-d1	GGGGACAAGTTTGTACAAAAAAGCAGGCTCCGAACTTCCTATTGCTAGAAG
JASnostop-gw-r1	GGGGACCACTTTGTACAAGAAAGCTGGGTGAGTATGGTGCCTTTGACGTAAC
JASA-gw-d1	GGGGACAAGTTTGTACAAAAAAGCAGGCTCCGAACTTCCTATTGCTGCAGCAGC
SHY2-gw-d1	GGGGACAAGTTTGTACAAAAAAGCAGGCTCCATGGATGAGTTTGTTAACCTC
SHY2nostop-gw-r1	GGGGACCACTTTGTACAAGAAAGCTGGGTGATACACCACAGCCTAAACC
SHY2-gw-r1	GGGGACCACTTTGTACAAGAAAGCTGGGTCATACACCACAGCCTAAACC
JASHY-d1	TACGTCAAAGGCACCATACATGGATGAGTTTGTTAACCTC
JASHY-r1	TGAGGTTAACAAACTCATCCATGTATGGTGCCTTTGACGT
DR5-gw-d1	GGGGACAAGTTTGTACAAAAAAGCAGGCTCCATGCCTGCAGGTCGACGGTAT
DR5-gw-r1	GGGGACCACTTTGTACAAGAAAGCTGGGTTTGTAATTGTAATTGTAAATAGT
ARF7-gw-d1	GGGGACAAGTTTGTACAAAAAAGCAGGCTCCATGAAAGCTCCTTCATCAAATGGAG
ARF7-gw-r1	GGGGACCACTTTGTACAAGAAAGCTGGGTCACCGGTTAAACGAAGTGGCTGAG
SeqL1	TCGCGTTAACGCTAGCATGGATCTC
SeqL2	GTAACATCAGAGATTTTGAGACAC
